# Impact of dietary selenium and blood concentration on liver function: a population-based study

**DOI:** 10.3389/fnut.2024.1415288

**Published:** 2024-07-17

**Authors:** Qiaoli Liang, Ruihua Huang, Ziming Peng, Menglong Zou

**Affiliations:** ^1^Doumen Qiaoli Hospital of Traditional Chinese Medicine, Zhuhai, Guangdong, China; ^2^Fangchenggang Hospital of Traditional Chinese Medicine, Fangchenggang, Guangxi, China; ^3^The First Hospital of Hunan University of Traditional Chinese Medicine, Changsha, Hunan, China

**Keywords:** dietary selenium intake, blood selenium concentration, liver function, cross-sectional study, NHANES

## Abstract

**Background:**

Evidence on the association between selenium and liver function parameters is limited and controversial.

**Methods:**

Data on dietary selenium intake, blood selenium concentration, and liver function parameters were obtained from the National Health and Nutrition Examination Survey (NHANES) 2017–2020. Associations between selenium (dietary intake and blood concentration) and liver function parameters [alanine aminotransferase (ALT), aspartate aminotransferase (AST), the ALT/AST ratio, gamma-glutamyl transferase (GGT), and alkaline phosphatase (ALP)] were assessed using multivariate linear regression models. Subgroup analyses and interaction tests were conducted to examine differences in associations according to age, gender, body mass index (BMI), diabetes, and physical activity.

**Results:**

The study included 6,869 participants after screening. The multivariate linear regression model revealed that dietary selenium intake was positively associated with ALT (β = 0.112, 95% CI = 0.041, 0.183) and the ALT/AST ratio (β = 0.002, 95% CI = 0.001, 0.004) after adjustment for covariates. Results of blood selenium concentration also showed that higher blood selenium levels were positively associated with ALT (β = 0.436, 95% CI = 0.308, 0.564), AST (β = 0.112, 95% CI = 0.015, 0.208), and the ALT/AST ratio (β = 0.012, 95% CI = 0.009, 0.015). However, ALP decreased with increasing blood selenium concentration (β = −0.207, 95% CI = −0.414, −0.000). In addition, we found significant differences in the effect of selenium on liver function parameters according to age, gender, and BMI.

**Conclusion:**

Dietary selenium intake and blood concentration affect liver function parameters. These findings suggest that further research is needed to explore these associations to promote liver health and disease prevention.

## 1 Introduction

The liver, being the largest metabolic organ in the human body, is essential for metabolism, detoxification, and biochemical synthesis (1). Liver injury can occur due to a variety of factors, including viral infections, excessive alcohol consumption, drug-induced hepatotoxicity, and metabolic disorders (2). Liver function tests (LFTs) are crucial for assessing liver status and the extent of injury. These tests include measurements of alanine aminotransferase (ALT), aspartate aminotransferase (AST), the ALT/AST ratio, gamma-glutamyl transferase (GGT), and alkaline phosphatase (ALP). The liver is not only sensitive to a variety of factors but is also intimately involved in the metabolism of numerous trace elements in the body (3, 4). In the multifaceted study of liver health, the role of trace minerals has received increasing attention (5–8).

Selenium is a vital micronutrient that is absorbed by the human body through the food chain, primarily from seafood, plant sources, and animal products (9). In the human body, selenium is not only a component of several important proteins, such as glutathione peroxidases and selenoprotein P, but also plays a role in antioxidant defense and maintaining cellular redox balance (10). Because of these important functions, selenium intake is closely linked to overall health, impacting cardiovascular disease (11, 12), immune function (13, 14), and thyroid health (15, 16). Blood selenium concentration is a crucial and reliable indicator of an individual’s overall selenium nutritional status. However, the relationship between selenium intake and blood concentration is not linear and is influenced by individual absorption, metabolism, and excretion processes (17). Both inadequate and excessive selenium intake can have adverse health effects. Therefore, understanding the relationship between dietary selenium intake and blood selenium concentration and how these factors influence human health, particularly in maintaining liver health, is critical for developing dietary guidelines and preventive strategies.

The role of selenium in liver health has received increasing attention due to its essential functions in enzymatic processes and antioxidant defense mechanisms (18). Selenium contributes significantly to the ability of the liver to mitigate oxidative stress, a known contributor to liver injury (19–21). Oxidative stress in the liver can be caused by several factors, including environmental toxins, metabolic imbalances, and chronic alcohol use, all of which can lead to liver cell damage and subsequent liver disease (22, 23). Research has shown that selenium deficiency may exacerbate liver injury by impairing the liver’s antioxidant defense system, thereby increasing susceptibility to oxidative damage (24, 25). However, the relationship between selenium and liver health is complex and dose-dependent, with both selenium deficiency and excess posing risks to liver function. This study aims to elucidate the relationship between dietary selenium intake, blood selenium concentration, and liver damage in US adults using data from the 2017–2020 National Health and Nutrition Examination Survey (NHANES). By examining these associations, this research aims to provide insight into the range of selenium intake and blood selenium concentrations for liver health and to contribute to the development of dietary guidelines and interventions aimed at preventing liver injury.

## 2 Materials and methods

### 2.1 Study population

**Figure 1:**
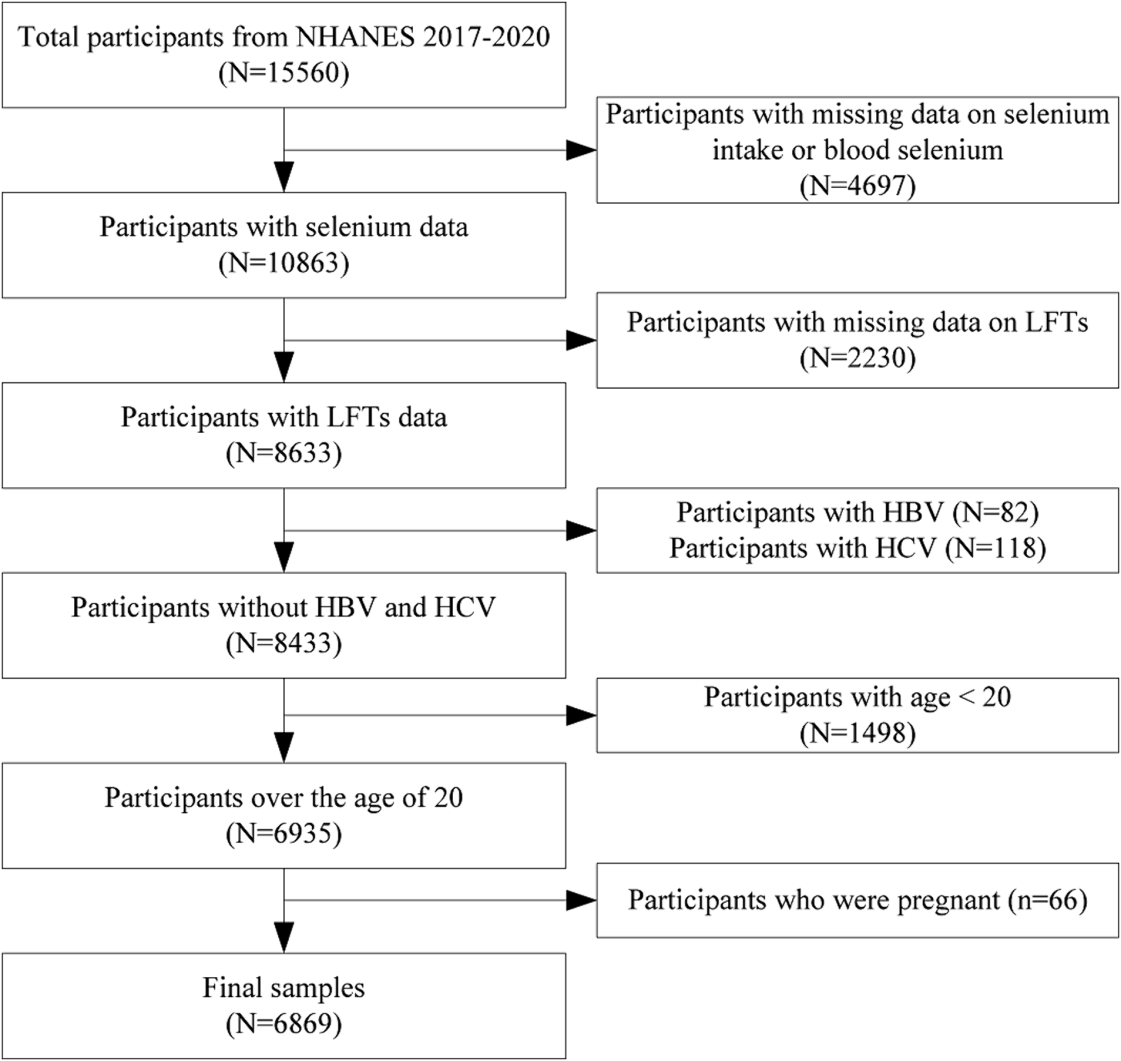
Flowchart of participants selection. NHANES, National health and nutrition examination survey; LFTs, Liver function tests; HBV, Hepatitis B virus; and HCV, Hepatitis C virus.

The NHANES is an annual, nationally representative survey conducted in the United States by the National Center for Health Statistics (NCHS) of the Centers for Disease Control and Prevention (CDC). NHANES has been utilizing an advanced multistage probability sampling design since 1999 to gather data biennially from a diverse, non-institutionalized section of the United States population. Ethical approval for NHANES was secured from the NCHS Research Ethics Review Board, and each participant willingly provided written informed consent. NHANES data from 2017 to 2020 were used in this study. From the initial pool of 15,560 eligible participants, several exclusions were made to ensure the integrity of the analysis: 4,697 individuals were excluded due to missing data on selenium intake or blood selenium levels, and 2,230 individuals were excluded due to missing LFTs results. The primary objective of this study is to investigate the impact of selenium on liver function in the general population. Including hepatitis-positive individuals could complicate the interpretation of results, as their abnormal liver function parameters may be attributable to the disease itself rather than selenium levels. Therefore, we excluded 82 participants who were positive for hepatitis B antibody and 118 participants who were positive for hepatitis C antibody. We excluded 1,498 individuals under the age of 20 because they are in a developmental stage, and their liver function parameters and selenium metabolism may differ significantly from adults, affecting the comparability and interpretability of the results. This exclusion also helps to enhance the homogeneity and data consistency of the study sample, and facilitates the analysis of the impact of educational level on the study results. We also excluded 66 individuals who reported being pregnant to refine the cohort for more accurate and relevant results. The study ultimately encompassed 6,869 participants (Figure 1).

### 2.2 Measurement of liver function

In NHANES, fasting blood samples were systematically collected from participants at a mobile examination center, ensuring consistency and standardization in the sample collection process. These samples were then immediately refrigerated and sent to a central laboratory for careful analysis. In the laboratory, serum liver function biomarkers were quantitatively assessed using the advanced Beckman Coulter DxC800 Synchron clinical system. ALT, primarily found in liver cells, is an enzyme involved in amino acid metabolism (26, 27). When liver cells are damaged or die, ALT is released into the blood. Elevated serum ALT levels are often seen as a sign of liver damage. AST, found in liver cells, cardiomyocytes, muscle cells, and other tissues, is not as liver-specific as ALT (26, 27). However, AST levels increase in response to liver injury. The ALT to AST ratio is used to differentiate between various types of liver disease. GGT and ALP are indicators of cholestasis (26, 27). Specifically, in this cross-sectional study, ALT, AST, ALT/AST ratio, GGT, and ALP were used to assess liver function impairment.

### 2.3 Measurement of dietary selenium intake and blood selenium level

The process for gathering dietary selenium data has been carefully designed to ensure accuracy and reliability. Dietary intake data were collected over two non-consecutive days, beginning with a face-to-face interview in a mobile examination center and followed by a telephone interview on a subsequent day. Participants were instructed to provide a detailed account of all food and beverages consumed in the previous 24 h, detailing specific meal contents and quantities. These details were then analyzed using the USDA’s Food and Nutrient Database for Dietary Studies, enabling an accurate estimation of selenium intake. To enhance individual dietary data accuracy, this study averaged selenium intake from two recall days, minimizing day-to-day dietary variation. In NHANES 2017–2020, whole blood selenium concentrations were measured using inductively coupled plasma mass spectrometry. Detailed measurements can be found in a previously published study (28).

### 2.4 Measurement of covariates

Covariates were identified as confounders based on previously published studies (29–31). Demographic characteristics, including gender, education, age, ethnicity, and family income-to-poverty ratio (PIR), were considered. Health-related factors included body mass index (BMI), alcohol consumption (at least 12 alcoholic beverages per year), smoking (at least 100 cigarettes in a lifetime), and physical activity (engaging in moderate or vigorous exercise or recreational activities weekly). Clinical factors included diabetes mellitus, fasting glucose, and total cholesterol (TC).

### 2.5 Statistical analysis

Dietary selenium intakes and blood selenium concentrations were divided into quartiles, with quartiles 1–4 representing progressively increasing intakes or concentrations. In the sample characteristics, continuous variables were expressed as means ± standard deviation, and categorical variables as count (number) and percentage. To compare means between groups, one-way ANOVA was used, while chi-squared tests were employed for percentages. Associations between selenium (dietary intake and blood concentration) and liver function parameters (ALT, AST, ALT/AST, GGT, and ALP) were calculated using multivariate linear regression models. The multivariate analysis utilized three models: crude model with no adjustment; Model 1 adjusted for age, gender, and race; and Model 2 included adjustments for the full range of covariates. Subgroup analyses and interaction tests were conducted to explore variations in the associations by age, gender, race, education, and BMI. *p* values less than 0.05 were considered statistically significant. The statistical analyses and graphical representations in this study were generated using R (version 4.1.3) and EmpowerStats (version 2.0).

## 3 Results

### 3.1 Baseline characteristics

Table 1 presents a comparison of baseline characteristics of participants across four quartiles of dietary selenium intake. A total of 6,869 participants with an average age of 50.788 ± 17.384 were included in this study, of which 48.62% were males and 51.38% were females. Notably, selenium intakes were higher than international recommendations and blood concentrations were also quite high. In addition, liver enzymes as well as cholesterol were within reference ranges. Blood selenium concentrations increased with each quartile of dietary selenium intake (*p* < 0.05). Significant differences were observed among the quartiles in age, gender, race, education level, family income-to-poverty ratio (PIR), BMI, fasting glucose, drinking habits, physical activity, and various liver function markers (all *p* < 0.05). Participants in the highest quartile were more likely to be male, consume alcohol, have above high school education, engage in physical activity, and have higher BMI, fasting glucose, ALT, AST, GGT, and ALT/AST levels, but lower ALP levels compared to those in the lowest quartile.

**Table 1:** Baseline characteristics of participants according to dietary selenium intake.

Variables	Dietary selenium intake (μg/day)				*p* value
	Q1 (<65.4) *N* = 1,717	Q2 (65.4–93.3) *N* = 1,717	Q3 (93.3–127.8) *N* = 1,715	Q4 (>127.8) *N* = 1,720	
Age, year, Mean (SD)	49.01 (17.70)	49.45 (17.90)	49.10 (17.16)	45.96 (16.02)	**<0.001**
Gender, *N* (%)					**<0.001**
Male	584 (34.01%)	687 (40.01%)	881 (51.37%)	1,188 (69.07%)	
Female	1,133 (65.99%)	1,030 (59.99%)	834 (48.63%)	532 (30.93%)	
Race, *N* (%)					**<0.001**
Mexican American	175 (10.19%)	205 (11.94%)	218 (12.71%)	231 (13.43%)	
Other Hispanic	202 (11.76%)	158 (9.20%)	168 (9.80%)	179 (10.41%)	
Non-Hispanic White	598 (34.83%)	671 (39.08%)	658 (38.37%)	593 (34.48%)	
Non-Hispanic Black	445 (25.92%)	442 (25.74%)	428 (24.96%)	423 (24.59%)	
Other race-including multi-racial	297 (17.30%)	241 (14.04%)	243 (14.17%)	294 (17.09%)	
Education level, *N* (%)					**<0.001**
Above high school	873 (50.84%)	986 (57.43%)	1,092 (63.67%)	1,058 (61.51%)	
Completed high school	391 (22.77%)	299 (17.41%)	267 (15.57%)	259 (15.06%)	
Less than high school	453 (26.38%)	432 (25.16%)	356 (20.76%)	403 (23.43%)	
Ratio of family income to poverty, Mean (SD)	2.81 (1.55)	3.10 (1.52)	3.35 (1.53)	3.21 (1.55)	**<0.001**
BMI, kg/m^2^, Mean (SD)	29.44 (7.36)	29.46 (6.93)	29.88 (6.90)	30.51 (7.69)	**<0.001**
Diabetes, *N* (%)					0.948
Yes	287 (16.72%)	260 (15.14%)	265 (15.45%)	239 (13.90%)	
No	1,393 (81.13%)	1,409 (82.06%)	1,389 (80.99%)	1,428 (83.02%)	
Borderline	37 (2.15%)	48 (2.80%)	61 (3.56%)	53 (3.08%)	
Fasting glucose, mg/dL, Mean (SD)	110.96 (20.45)	110.48 (20.27)	112.75 (24.68)	112.63 (25.18)	**0.004**
Drink, *N* (%)					**<0.001**
Yes	647 (37.68%)	755 (43.97%)	839 (48.92%)	896 (52.09%)	
No	1,070 (62.32%)	962 (56.03%)	876 (51.08%)	824 (47.91%)	
Smoke, *N* (%)					0.105
Yes	693 (40.36%)	702 (40.89%)	710 (41.40%)	772 (44.88%)	
No	1,024 (59.64%)	1,015 (59.11%)	1,005 (58.60%)	948 (55.12%)	
Physical activity, *N* (%)					**<0.001**
Yes	723 (42.11%)	793 (46.19%)	864 (50.38%)	913 (53.08%)	
No	994 (57.89%)	924 (53.81%)	851 (49.62%)	807 (46.92%)	
ALT, U/L, Mean (SD)	20.27 (14.59)	20.93 (14.38)	22.98 (16.46)	25.13 (16.64)	**<0.001**
ALP, U/L, Mean (SD)	77.18 (25.49)	75.31 (24.40)	73.64 (25.44)	75.44 (23.88)	**0.016**
AST, U/L, Mean (SD)	20.55 (10.12)	21.03 (12.49)	22.18 (12.17)	22.15 (9.20)	**<0.001**
GGT, U/L, Mean (SD)	27.05 (32.73)	27.39 (31.75)	29.35 (43.50)	31.61 (35.50)	**<0.001**
ALT/AST, Mean (SD)	0.96 (0.34)	0.98 (0.33)	1.01 (0.35)	1.11 (0.37)	**<0.001**
TC, mmol/L, Mean (SD)	4.89 (1.06)	4.88 (1.05)	4.86 (1.01)	4.79 (1.05)	0.192
Blood selenium concentration, μg/L, Mean (SD)	187.66 (26.07)	187.52 (28.38)	188.87 (26.45)	190.76 (27.99)	**<0.001**

Significant results highlighted in bold. BMI, Body mass index; TC, Total cholesterol; ALT, Alanine aminotransferase; AST, Aspartate aminotransferase; GGT, Gamma-glutamyl transferase; ALP, Alkaline phosphatase.

Table 2 compares the baseline characteristics of participants according to four quartiles of blood selenium concentration. With increasing dietary selenium intake, the quartiles of the participants’ blood selenium concentrations increased (*p* < 0.05). Significant differences in age, gender, race, education level, fasting glucose, drinking habits, physical activity, ALT, AST, GGT, ALT/AST, and TC (all *p* < 0.05). Participants in the highest quartile were more likely to be male, to have above high school education, to be physically active, and to have higher levels of fasting glucose, ALT, AST, GGT, ALT/AST, and TC, compared to participants in the lowest quartile.

**Table 2:** Baseline characteristics of participants according to blood selenium concentration.

Variables	Blood selenium concentration (μg/L)				*p* value
	Q1 (<168.8)	Q2 (168.8–183.75)	Q3 (183.75–200.55)	Q4 (>200.55)	
	*N* = 1,716	*N* = 1,717	*N* = 1,718	*N* = 1,718	
Age, year, Mean (SD)	49.29 (18.08)	47.50 (17.31)	47.91 (17.07)	48.78 (16.63)	**0.014**
Gender, *N* (%)					**<0.001**
Male	758 (44.17%)	820 (47.76%)	836 (48.66%)	926 (53.90%)	
Female	958 (55.83%)	897 (52.24%)	882 (51.34%)	792 (46.10%)	
Race, *N* (%)					**<0.001**
Mexican American	157 (9.15%)	212 (12.35%)	242 (14.09%)	218 (12.69%)	
Other Hispanic	189 (11.01%)	185 (10.77%)	159 (9.25%)	174 (10.13%)	
Non-Hispanic White	604 (35.20%)	621 (36.17%)	666 (38.77%)	629 (36.61%)	
Non-Hispanic Black	559 (32.58%)	470 (27.37%)	367 (21.36%)	342 (19.91%)	
Other race-including multi-racial	207 (12.06%)	229 (13.34%)	284 (16.53%)	355 (20.66%)	
Education level, *N* (%)					**<0.001**
Above high school	916 (53.38%)	993 (57.83%)	1,051 (61.18%)	1,049 (61.06%)	
Completed high school	459 (26.75%)	430 (25.04%)	368 (21.42%)	387 (22.53%)	
Less than high school	341 (19.87%)	294 (17.12%)	299 (17.40%)	282 (16.41%)	
Ratio of family income to poverty, Mean (SD)	3.05 (1.60)	3.15 (1.54)	3.16 (1.57)	3.14 (1.50)	0.173
BMI, kg/m^2^, Mean (SD)	29.65 (7.64)	29.83 (7.28)	29.97 (7.01)	29.87 (7.10)	0.662
Diabetes, *N* (%)					0.444
Yes	266 (15.50%)	247 (14.39%)	249 (14.49%)	289 (16.82%)	
No	1,407 (81.99%)	1,419 (82.64%)	1,418 (82.54%)	1,375 (80.03%)	
Borderline	43 (2.51%)	51 (2.97%)	51 (2.97%)	54 (3.14%)	
Fasting glucose, mg/dL, Mean (SD)	112.00 (27.17)	110.48 (18.44)	110.91 (20.36)	113.50 (25.04)	**<0.001**
Drink, *N* (%)					**<0.001**
Yes	728 (42.42%)	798 (46.48%)	838 (48.78%)	773 (44.99%)	
No	988 (57.58%)	919 (53.52%)	880 (51.22%)	945 (55.01%)	
Smoke, *N* (%)					0.056
Yes	737 (42.95%)	726 (42.28%)	700 (40.75%)	714 (41.56%)	
No	979 (57.05%)	991 (57.72%)	1,018 (59.25%)	1,004 (58.44%)	
Physical activity, *N* (%)					**0.021**
Yes	741 (43.18%)	825 (48.05%)	868 (50.52%)	859 (50.00%)	
No	975 (56.82%)	892 (51.95%)	850 (49.48%)	859 (50.00%)	
ALT, U/L, Mean (SD)	19.55 (12.01)	21.81 (15.33)	23.27 (15.48)	24.33 (18.20)	**<0.001**
ALP, U/L, Mean (SD)	75.93 (28.87)	74.44 (23.69)	76.96 (24.17)	74.07 (22.97)	0.235
AST, U/L, Mean (SD)	20.80 (10.00)	21.30 (11.02)	21.74 (9.63)	22.06 (13.11)	**<0.001**
GGT, U/L, Mean (SD)	28.75 (45.82)	27.06 (31.40)	29.21 (34.06)	30.49 (34.67)	**0.044**
ALT/AST, Mean (SD)	0.93 (0.32)	0.99 (0.34)	1.04 (0.36)	1.08 (0.37)	**<0.001**
TC, mmol/L, Mean (SD)	4.59 (0.93)	4.83 (1.05)	4.86 (1.02)	5.06 (1.09)	**<0.001**
Dietary selenium intake, μg/day, Mean (SD)	157.12 (10.20)	176.47 (4.26)	191.56 (4.86)	220.48 (25.55)	**<0.001**

Significant results highlighted in bold. BMI, Body mass index; TC, Total cholesterol; ALT, Alanine aminotransferase; AST, Aspartate aminotransferase; GGT, Gamma-glutamyl transferase; and ALP, Alkaline phosphatase.

### 3.2 Relationship between dietary selenium intake and liver function parameters

**Figure 2:**
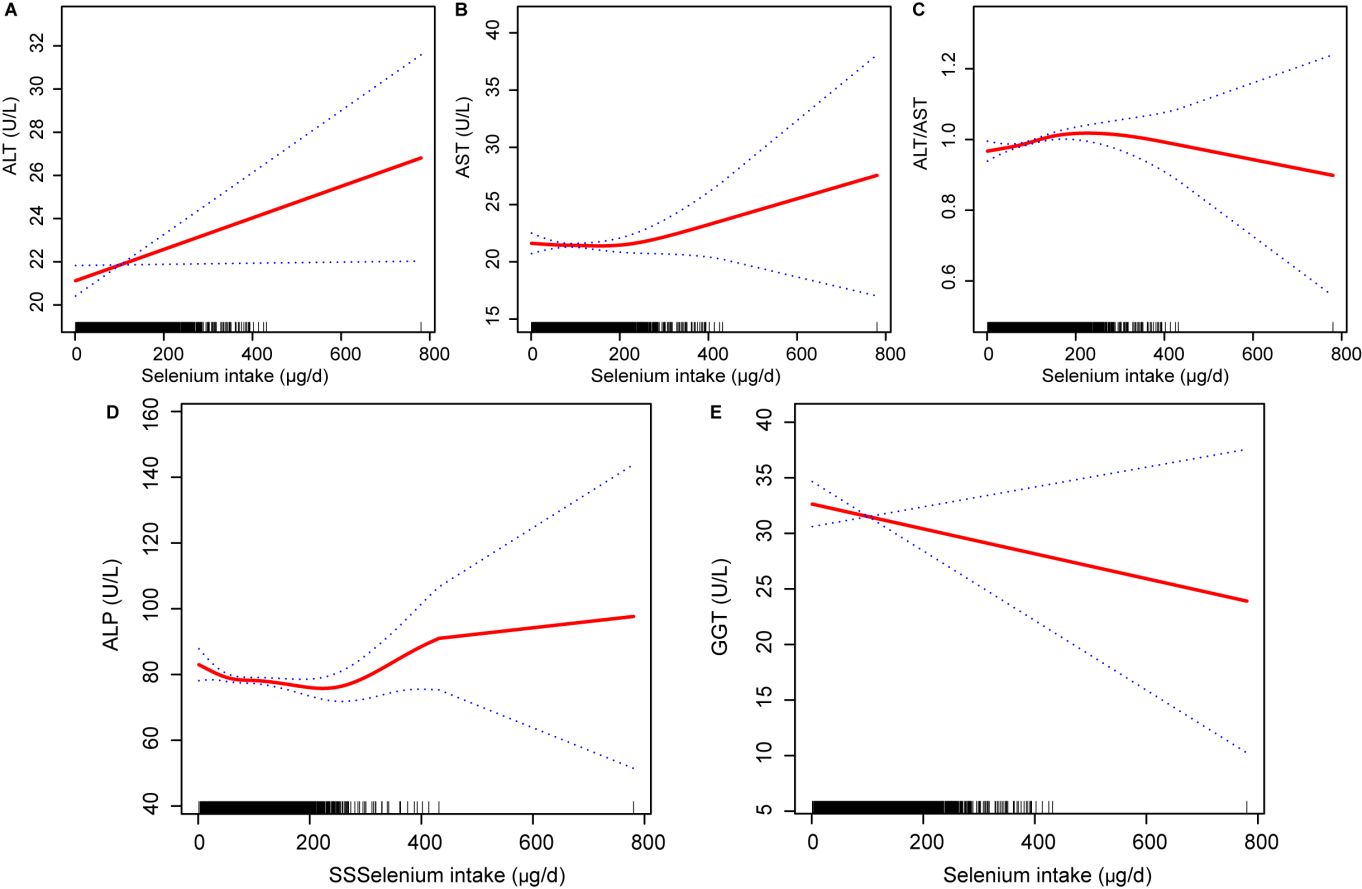
Relationship between dietary selenium intake **(A)** ALT, **(B)** AST, **(C)** ALT/AST, **(D)** ALP; and **(E)** GGT. ALT, Alanine aminotransferase; AST, Aspartate aminotransferase; GGT, Gamma-glutamyl transferase; ALP, Alkaline phosphatase.

To better observe the relationship between selenium intake and liver function parameters, the unit of selenium intake was converted to 10 μg per day. The results of multivariate linear regression analyses examining the association between dietary selenium intake and liver function parameters are shown in Table 3. The present study found a positive association between dietary selenium intake and ALT and the ALT/AST ratio whatever the model. There was no robust association with other liver function parameters. The effect of selenium intake on liver function parameters was illustrated through a smoothed curve fit (Figure 2). Dietary selenium intake was then categorized into quartile variables to examine the linear relationship (Table 4). In model 2, higher selenium intake was associated with higher effect values for ALT and ALT/AST.

**Table 3:** Multiple linear regression associations of dietary selenium intake (continuous) with liver function parameters.

Exposure	Outcome	β (95%CI), *p* value		
		Crude	Model 1	Model 2
Selenium intake	ALT	0.416 (0.345, 0.488) **<0.001**	0.163 (0.090, 0.236) **<0.001**	0.112 (0.041, 0.183) **0.002**
	AST	0.162 (0.111, 0.213) **<0.001**	0.058 (0.005, 0.112) **0.032**	0.049 (−0.005, 0.102) 0.075
	ALT/AST	0.010 (0.009, 0.012) **<0.001**	0.004 (0.002, 0.006) **<0.001**	0.002 (0.001, 0.004) **0.005**
	ALP	−0.135 (−0.249, −0.020) **0.021**	−0.079 (−0.197, 0.039) 0.190	−0.039 (−0.153, 0.076) 0.507
	GGT	0.266 (0.098, 0.433) **0.002**	−0.048 (−0.223, 0.126) 0.588	−0.060 (−0.232, 0.111) 0.492

Crude: no covariates were adjusted. Model 1: age, gender, and race were adjusted. Model 2: age, gender, race, education, ratio of family income to poverty, diabetes, BMI, drink, smoke, physical activity, and TC were adjusted. Significant results highlighted in bold. BMI, Body mass index; TC, Total cholesterol, ALT, Alanine aminotransferase; AST, Aspartate aminotransferase; GGT, Gamma-glutamyl transferase; and ALP, Alkaline phosphatase.

**Table 4:** Multiple linear regression associations of dietary selenium intake (quartile) with liver function parameters.

LFTs	Model	Dietary selenium intake (quartile)				*p* for trend
		Q1	Q2 β (95% CI)	Q3 β (95% CI)	Q4 β (95% CI)	
ALT	Crude	Reference	0.660 (−0.417, 1.738)	2.710 (1.651, 3.769)	4.862 (3.799, 5.924)	**<0.001**
	Model 1	Reference	0.046 (−0.995, 1.087)	1.102 (0.069, 2.134)	1.589 (0.524, 2.654)	**<0.001**
	Model 2	Reference	−0.064 (−1.073, 0.945)	0.660 (−0.348, 1.668)	0.897 (−0.140, 1.934)	**0.039**
AST	Crude	Reference	0.484 (−0.282, 1.250)	1.636 (0.882, 2.389)	1.606 (0.851, 2.361)	**<0.001**
	Model 1	Reference	0.198 (−0.562, 0.957)	0.922 (0.169, 1.675)	0.219 (−0.558, 0.996)	0.277
	Model 2	Reference	0.136 (−0.622, 0.893)	0.809 (0.052, 1.565)	0.113 (−0.666, 0.891)	0.439
ALT/AST	Crude	Reference	0.021 (−0.003, 0.045)	0.056 (0.032, 0.079)	0.149 (0.126, 0.173)	**<0.001**
	Model 1	Reference	0.006 (−0.018, 0.029)	0.016 (−0.007, 0.039)	0.068 (0.045, 0.092)	**<0.001**
	Model 2	Reference	0.003 (−0.018, 0.024)	0.001 (−0.021, 0.022)	0.042 (0.021, 0.064)	**<0.001**
ALP	Crude	Reference	−1.873 (−3.584, −0.161)	−3.536 (−5.218, −1.853)	−1.734 (−3.421, −0.047)	**0.016**
	Model 1	Reference	−1.831 (−3.509, −0.153)	−3.240 (−4.905, −1.575)	−0.735 (−2.452, 0.982)	0.220
	Model 2	Reference	−0.818 (−2.443, 0.808)	−1.858 (−3.482, −0.234)	−0.050 (−1.721, 1.620)	0.704
GGT	Crude	Reference	0.337 (−2.174, 2.848)	2.301 (−0.168, 4.770)	4.557 (2.080, 7.033)	**<0.001**
	Model 1	Reference	−0.556 (−3.043, 1.931)	0.271 (−2.196, 2.738)	0.716 (−1.828, 3.260)	0.453
	Model 2	Reference	−0.198 (−2.635, 2.239)	0.405 (−2.029, 2.840)	0.741 (−1.764, 3.246)	0.476

Crude: no covariates were adjusted. Model 1: age, gender, and race were adjusted. Model 2: age, gender, race, education, ratio of family income to poverty, diabetes, BMI, drink, smoke, physical activity, and TC were adjusted. Significant results highlighted in bold. BMI, Body mass index; TC, Total cholesterol; ALT, Alanine aminotransferase; AST, Aspartate aminotransferase; GGT, Gamma-glutamyl transferase; and ALP, Alkaline phosphatase.

### 3.3 Relationship between blood selenium concentration and liver function parameters

**Figure 3:**
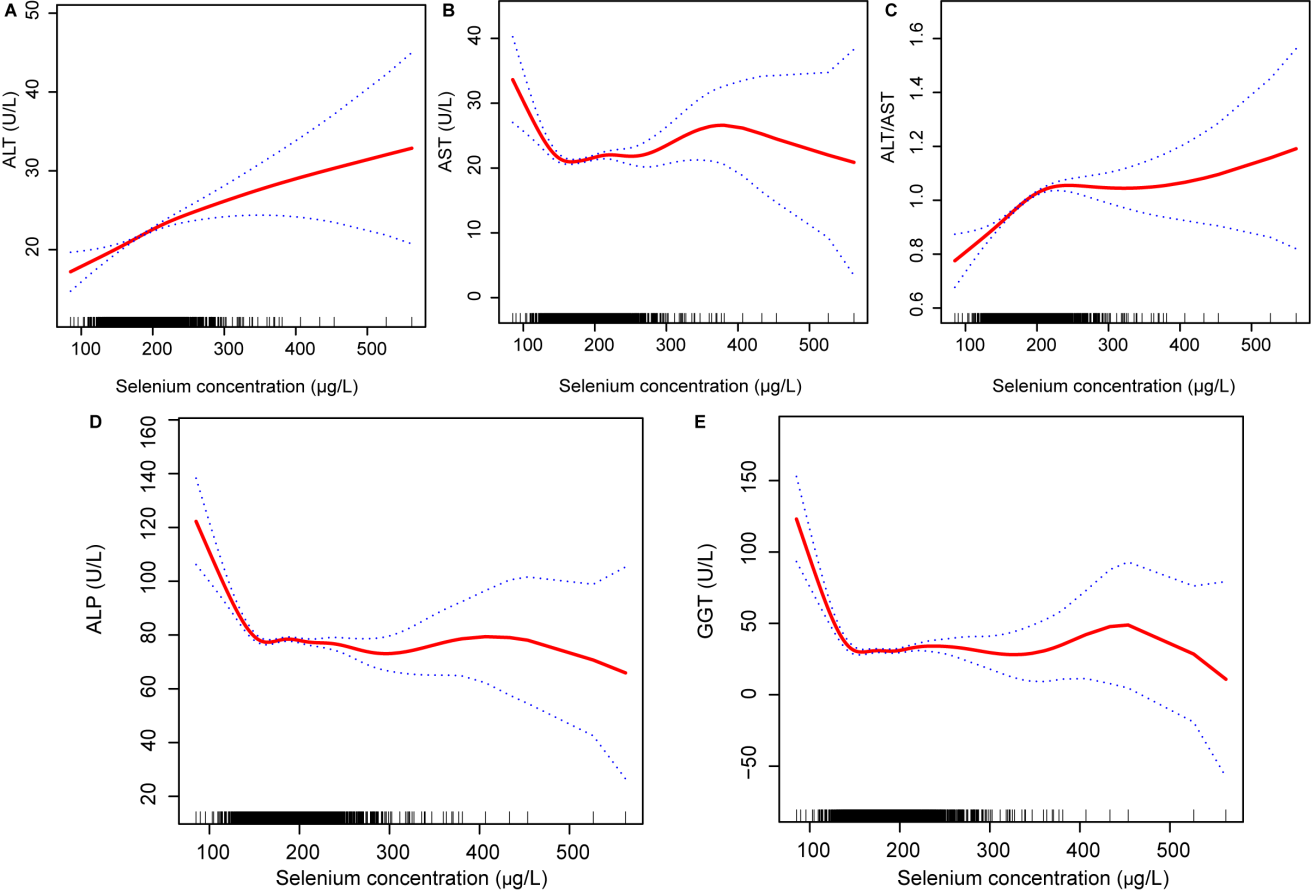
Relationship between blood selenium concentration **(A)** ALT, **(B)** AST, **(C)** ALT/AST, **(D)** ALP; and **(E)** GGT. ALT, Alanine aminotransferase; AST, Aspartate aminotransferase; GGT, Gamma-glutamyl transferase; and ALP, Alkaline phosphatase.

Similarly, blood selenium concentrations were converted to 10 μg/L. Results from multivariate linear regression analyses examining the association between blood selenium concentration and liver function parameters are presented in Table 5. Consistent with the findings for dietary selenium intake, blood selenium concentrations were positively correlated with ALT and the ALT/AST ratio. Additionally, blood selenium concentrations were positively correlated with AST and negatively correlated with ALP in model 2. The effect of blood selenium on liver function parameters was illustrated through a smoothed curve fit (Figure 3). Blood selenium concentration was then categorized into quartile variables to examine the linear relationship (Table 6). In model 2, similar trend test results were observed for ALT and the ALT/AST ratio (*p* for trend <0.001), but not for AST and ALP.

**Table 5:** Multiple linear regression associations of blood selenium concentration (continuous) with liver function parameters.

Exposure	Outcome	β (95%CI), *P* value		
		Crude	Model 1	Model 2
Blood selenium	ALT	0.644 (0.509, 0.780) **<0.001**	0.527 (0.397, 0.658) **<0.001**	0.436 (0.308, 0.564) **<0.001**
	AST	0.205 (0.109, 0.301) **<0.001**	0.154 (0.058, 0.250) **0.002**	0.112 (0.015, 0.208) **0.023**
	ALT/AST	0.017 (0.014, 0.020) **<0.001**	0.014 (0.011, 0.017) **<0.001**	0.012 (0.009, 0.015) **<0.001**
	ALP	−0.228 (−0.443, −0.013) **0.038**	−0.211 (−0.423, 0.000) 0.051	−0.207 (−0.414, −0.000) **0.049**
	GGT	0.215 (−0.101, 0.530) 0.182	0.099 (−0.214, 0.413) 0.535	−0.109 (−0.420, 0.201) 0.489

Crude: no covariates were adjusted. Model 1: age, gender, and race were adjusted. Model 2: age, gender, race, education, ratio of family income to poverty, diabetes, BMI, drink, smoke, physical activity, and TC were adjusted. Significant results highlighted in bold. BMI, Body mass index; TC, Total cholesterol; ALT, Alanine aminotransferase; AST, Aspartate aminotransferase; GGT, Gamma-glutamyl transferase; and ALP, Alkaline phosphatase.

**Table 6:** Multiple linear regression associations of blood selenium concentration (quartile) with liver function parameters.

LFTs	Model	Blood selenium concentration (quartile)				*p* for trend
		Q1	Q2 β (95% CI)	Q3 β (95% CI)	Q4 β (95% CI)	
ALT	Crude	Reference	2.263 (1.161, 3.364)	3.723 (2.643, 4.803)	4.779 (3.711, 5.848)	**<0.001**
	Model 1	Reference	1.947 (0.889, 3.004)	3.191 (2.153, 4.230)	3.772 (2.742, 4.802)	**<0.001**
	Model 2	Reference	1.468 (0.441, 2.495)	2.591 (1.579, 3.602)	2.783 (1.771, 3.795)	**<0.001**
AST	Crude	Reference	0.501 (−0.282, 1.284)	0.942 (0.174, 1.710)	1.256 (0.496, 2.016)	**<0.001**
	Model 1	Reference	0.432 (−0.341, 1.206)	0.784 (0.024, 1.544)	0.862 (0.108, 1.616)	**0.018**
	Model 2	Reference	0.217 (−0.556, 0.990)	0.504 (−0.257, 1.266)	0.432 (−0.330, 1.194)	0.209
ALT/AST	Crude	Reference	0.064 (0.039, 0.089)	0.107 (0.082, 0.131)	0.145 (0.121, 0.169)	**<0.001**
	Model 1	Reference	0.054 (0.031, 0.078)	0.091 (0.068, 0.114)	0.119 (0.096, 0.141)	**<0.001**
	Model 2	Reference	0.044 (0.023, 0.066)	0.078 (0.057, 0.099)	0.097 (0.075, 0.118)	**<0.001**
ALP	Crude	Reference	−1.495 (−3.242, 0.253)	1.022 (−0.692, 2.735)	−1.866 (−3.561, −0.170)	0.235
	Model 1	Reference	−1.008 (−2.718, 0.703)	1.622 (−0.058, 3.303)	−1.448 (−3.115, 0.218)	0.455
	Model 2	Reference	−0.952 (−2.608, 0.704)	1.990 (0.359, 3.621)	−1.841 (−3.473, −0.209)	0.259
GGT	Crude	Reference	−1.694 (−4.260, 0.872)	0.456 (−2.060, 2.972)	1.740 (−0.749, 4.230)	**0.044**
	Model 1	Reference	−1.550 (−4.083, 0.984)	0.509 (−1.980, 2.997)	0.978 (−1.490, 3.447)	0.167
	Model 2	Reference	−2.733 (−5.219, −0.248)	−0.823 (−3.271, 1.625)	−1.525 (−3.976, 0.925)	0.590

Crude: no covariates were adjusted. Model 1: age, gender, and race were adjusted. Model 2: age, gender, race, education, ratio of family income to poverty, diabetes, BMI, drink, smoke, physical activity, and TC were adjusted. Significant results highlighted in bold. BMI, Body mass index; TC, Total cholesterol; ALT, Alanine aminotransferase; AST, Aspartate aminotransferase; GGT, Gamma-glutamyl transferase; and ALP, Alkaline phosphatase.

### 3.4 Subgroup analysis

**Figure 4:**
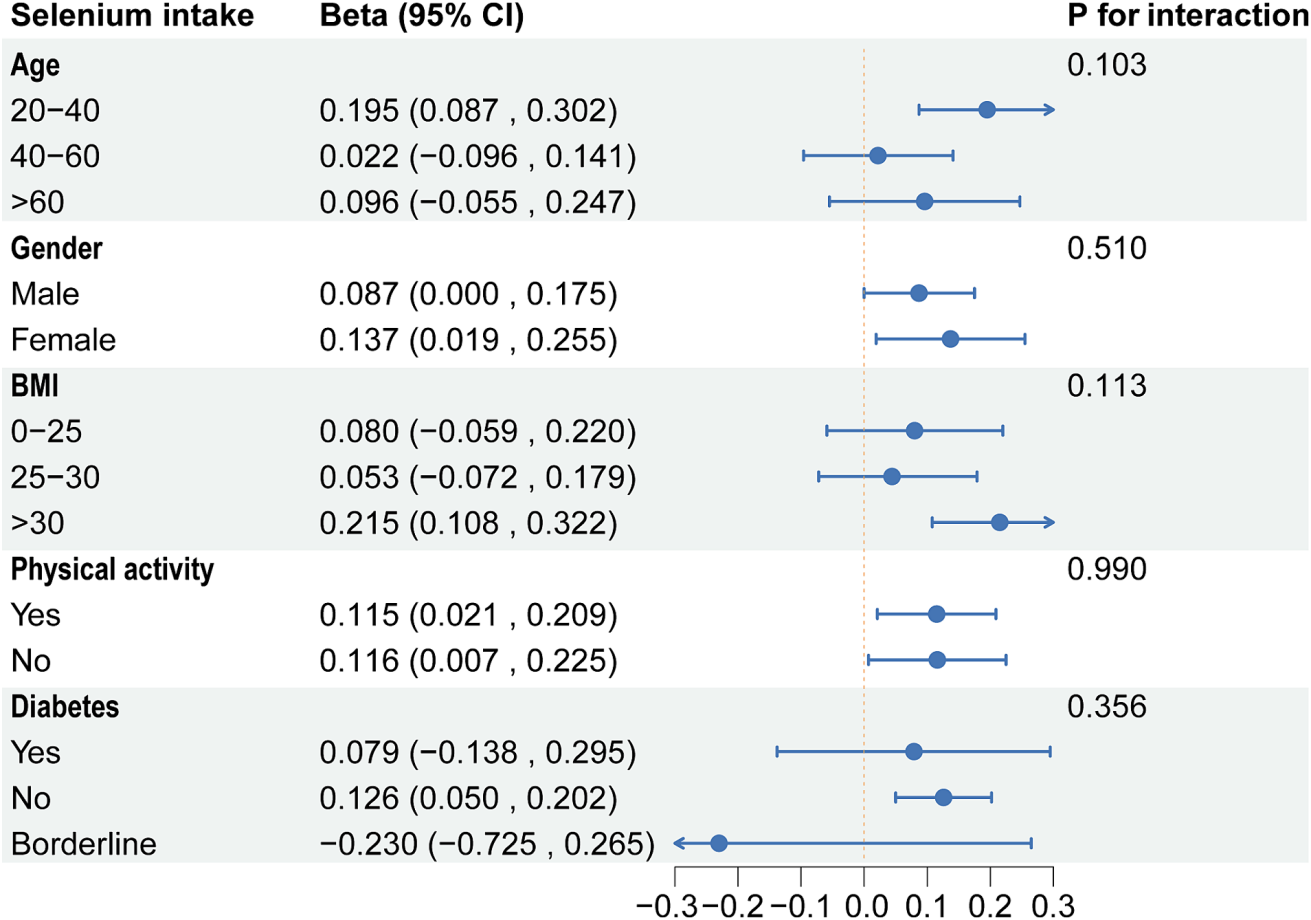
Subgroup analysis of the association between dietary selenium intake and ALT. ALT, Alanine aminotransferase.

**Figure 5:**
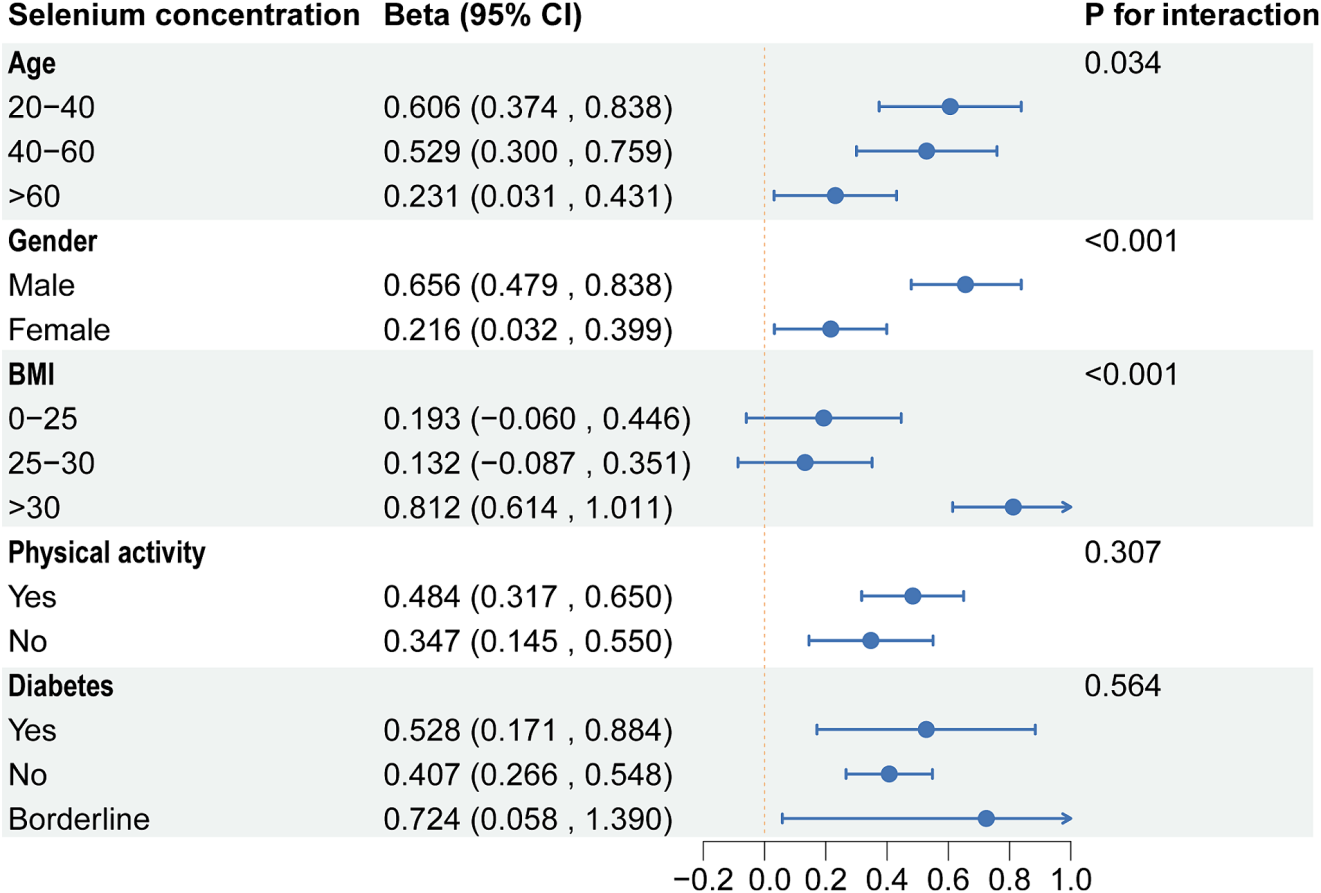
Subgroup analysis of the association between blood selenium concentration and ALT. ALT, Alanine aminotransferase.

Based on the above results, we found correlations between dietary selenium and blood concentrations with ALT. Subgroup analyses further explored the effects of age, gender, BMI, diabetes, and physical activity on these associations. The present results showed that correlations between selenium intake and blood concentrations with ALT were not consistent. Significant associations were found between dietary selenium and ALT in participants aged 20–40 years, female, BMI > 30, non-diabetic, and with or without physical activity (Figure 4). The results of interaction tests indicated that there were no statistically significant variations in the association between dietary selenium intake and ALT across different stratifications, implying that age, gender, BMI, diabetes, and physical activity did not have a significant impact on this positive correlation. However, interaction tests revealed that the association between selenium blood concentrations and ALT levels is significantly influenced by age, gender, and BMI stratifications (Figure 5). Higher selenium concentrations were associated with a significant increase in ALT levels across all age and gender stratifications, with a more pronounced effect in individuals aged 20–40 and in males. The increase in ALT levels was also significant in obese individuals. In addition, although the interaction test for physical activity and diabetes status was not significant, higher selenium concentrations still significantly increased ALT levels in all stratifications of physical activity and diabetes status.

## 4 Discussion

In this cross-sectional population-based study, dietary selenium intake was consistently positively associated with ALT and the ALT/AST ratio before and after adjustment for covariates. In addition, results indicated that high blood selenium was positively associated with ALT, AST, and ALT/AST ratio. However, ALP levels decreased with increasing blood selenium concentrations. In conclusion, this cross-sectional study demonstrated that dietary selenium intake and blood selenium concentration affect liver function parameters. Selenium is an essential trace element, and adequate intake is crucial for maintaining liver health. However, the use of selenium supplements should be approached with caution in light of our findings. While selenium has health benefits, excessive intake can lead to adverse effects, including potential effects on liver function. Therefore, clinicians should consider patients’ total selenium intake when recommending supplements to avoid overconsumption.

The role of selenium as a micronutrient essential for various bodily functions has been well-documented over the decades. However, supplementation, especially in the context of the Selenium and Vitamin E Cancer Prevention Trial, has raised concerns due to adverse outcomes like increased incidence of type 2 diabetes and prostate cancer (32, 33). Reports from the 1930s about selenium’s potential hepatotoxic effects in overexposure cases set a precedent for careful selenium dosage consideration in supplementation practices (34). In a recent study, Li et al. (35) associated blood selenium with elevated AST in 15,328 samples from NHANES 2011–2018, which is consistent with our findings. However, they did not explore dietary selenium intake, subgroup analyses, or BMI’s impact on selenium metabolism. Yang et al.’s cross-sectional study in China with 8,550 participants found significant ALT and AST increases with rising plasma selenium concentrations (36). They observed a linear correlation between plasma selenium and ALT and AST when converting plasma selenium concentrations into a categorical variable. Moreover, our results align with Longnecker et al. (37), who noted a positive correlation between selenium intake and ALT levels in United States selenium-enriched regions. Such corroborative findings reinforce the theoretical link between selenium and liver health.

The major antioxidant effects of selenium are mediated by selenium-dependent glutathione peroxidase (GPx) and selenoproteins (38–40). Maximum selenoprotein levels in the blood were in the range of 70–90 μg/L (41). The increase in blood selenium above these levels does not reflect an increase in selenoprotein levels but primarily reflects non-specific binding of selenomethionine to albumin instead of methionine (41). The mean serum selenium level of the participants was 186.11 μg/L, and the increase in selenium levels may have resulted in the non-specific binding of selenomethionine. A previous report also showed that the dietary selenium intake of United States adults far exceeds the amount of selenium needed to achieve maximal blood selenoprotein levels (42). Adequate selenium intake regulates the activity of selenoproteins, such as thioredoxin reductase (TrxR), thereby enhancing the body’s antioxidant capacity and mitigating the adverse effects of oxidative stress (43). TrxR and GPx, as core components of the thioredoxin (Trx) and glutathione (GSH) systems, constitute key thiol/selenol-dependent antioxidant systems. These systems maintain redox balance within the body and protect cells from oxidative damage (44–46). Selenium deficiency leads to decreased TrxR activity (47, 48). Compared to low selenium levels, selenium supplementation increases TrxR activity but stabilizes at normal levels. Notably, excessive selenium does not further increase TrxR activity. Conversely, in an acetaminophen (APAP)-induced liver injury model, selenium-enriched mice exhibit more severe liver damage than mice with normal selenium levels (49). Typical selenium compounds, such as sodium selenite, have been widely used at high concentrations to inhibit tumor cell growth (50). In mammals, selenite is reduced by TrxR to produce hydrogen selenide, which is highly reactive with oxygen, thereby generating reactive oxygen species (ROS). Selenomethionine, an organic selenium compound, is one of the predominant forms of selenium in nature. It is metabolized through various pathways within cells. Compared to selenite, higher selenium doses generates hydrogen selenide as a metabolic product, leading to oxidative stress (51). In summary, excessive selenium significantly increases oxidative stress levels within hepatocytes, causing cellular damage, which may be the underlying biological mechanism for the observed elevation in ALT levels induced by excessive selenium.

In subgroup analyses, significant associations were found between dietary selenium and ALT in participants aged 20–40 years, female, BMI >30, and non-diabetes. The results of interaction tests indicated that there were no statistically significant variations in the association between dietary selenium intake and ALT across different stratifications. However, interaction tests revealed that the association between selenium blood concentrations and ALT levels was significantly influenced by age, gender, and BMI stratifications. Higher selenium concentrations were associated with a significant increase in ALT levels across all age and gender stratifications, with a more pronounced effect in individuals aged 20–40 and in males. The increase in ALT levels was also significant in obese individuals. Adults between the ages of 20 and 40 are in a period of high metabolic activity, which may make them more sensitive to selenium intake, especially if the intake exceeds the body’s needs, which may have adverse effects on liver function (52). Individuals with higher body weight, especially those who are obese, may have altered selenium distribution and action owing to metabolic activities in adipose tissue, which could amplify adverse effects on liver function (53). These findings underscore the need to consider individual variability in research on the relationship between selenium intake and liver health. Future strategies and guidelines should be personalized, accounting for the unique needs and risks of diverse populations to ensure beneficial selenium intake levels for liver health.

In this cross-sectional study, the effects of dietary selenium intake on ALT, ALT/AST, and GGT were consistent with the results for blood selenium concentration. However, the findings regarding AST and ALP were inconsistent. Methodologically, the differences might be due to the nature of the data collection. Dietary selenium intake data is typically based on 24-h dietary recalls, which can be affected by recall bias and reporting accuracy. Blood selenium levels, on the other hand, reflect the actual storage and utilization of selenium in the body, which may have a time lag and individual variability. Additionally, the bioavailability of dietary selenium can vary depending on the type of food and an individual’s absorption capacity, leading to different impacts of dietary selenium intake and blood selenium levels on AST and ALP. Biologically, the metabolism and function of selenium are complex and varied. Selenium primarily exerts its antioxidant effects through GPx and selenoprotein P, which have different expression and activity levels in various tissues, possibly resulting in differential impacts on liver enzymes. ALT is predominantly found in hepatocytes, while AST is expressed in multiple tissues including the liver, heart, and muscles, which might explain the different mechanisms of selenium’s effects on ALT and AST. Furthermore, selenium might influence ALP levels through its role in regulating apoptosis and inflammatory responses, which can vary depending on an individual’s genetic background and health status.

While our study has revealed associations between blood selenium levels, dietary selenium intake, and liver function parameters, it is important to further investigate the different forms of selenium in the blood and their varying effects on liver function. Selenium exists in multiple forms in the blood, including inorganic selenium (such as selenite and selenate) and organic selenium (such as selenomethionine and selenocysteine). These different forms of selenium have distinct biological activities and metabolic pathways, which may impact liver function in various ways. For instance, organic forms of selenium like selenomethionine can be non-specifically incorporated into body proteins as a long-term selenium storage, whereas inorganic selenium is mainly involved in the synthesis of selenoproteins like GPx. These different forms of selenium may differentially affect antioxidant capacity, cellular protection, and inflammatory responses, thus exhibiting varying effects on liver function parameters. Therefore, future studies should consider selenium speciation analysis to differentiate between these various forms of selenium and their respective impacts on liver function. This will provide a more comprehensive and detailed understanding of the biological roles of selenium and its mechanisms in liver function regulation, thereby offering a scientific basis for developing more precise public health strategies.

This study assessed the impact of dietary selenium intake and blood selenium concentration on liver function parameters among US adults using the latest NHANES dataset. A key strength of this study is its large sample size, coupled with a detailed characterization of the study population. Additionally, the adjustment for covariates enhances the reliability of results. Finally, we performed subgroup analyses and interaction tests to explore differences in these associations by age, gender, race, education level, and BMI.

Although this study provided a comprehensive analysis of dietary selenium intake and blood selenium concentration’s effects on liver function, it has several limitations. First, blood selenium concentration, a direct biomarker of exposure, can be influenced by short-term dietary changes, individual metabolic differences, and physiological conditions, potentially not reflecting long-term intake accurately. Additionally, the relationship between dietary intake and blood concentration can be complicated by individual differences in absorption and metabolism. Second, despite adjusting for numerous potential confounders, the study cannot eradicate all confounding biases. For instance, dietary records and questionnaires depend on self-reporting, susceptible to recall or reporting bias. Furthermore, this observational study identified associations between dietary selenium intake, blood concentrations, and liver function, but it cannot establish causality. Future research should confirm these associations via prospective or interventional studies. Third, despite this study exploring the association between selenium levels and various liver function indicators (such as ALT, AST, ALP, and GGT), we did not include bilirubin in our analysis. Bilirubin is an important indicator of liver metabolic capacity and biliary system patency, and its levels are influenced by multiple factors including hemolysis, liver metabolic function, and biliary tract patency. While ALT, AST, ALP, and GGT are also affected by various factors, the metabolic pathway of bilirubin is more complex and involves additional potential confounders. Therefore, we chose not to include bilirubin in the analysis for this study. We acknowledge that this decision introduces certain limitations, making the interpretation of selenium’s impact on overall liver function less comprehensive. Future research should incorporate bilirubin into the analysis to more accurately assess the multifaceted effects of selenium on liver function. Lastly, given that the NHANES database does not provide the exact dates of dietary recall interviews and blood sample collections, we cannot directly analyze the temporal relationship between these two variables. This hinders our ability to assess the time-dependent effects of dietary selenium intake and blood selenium concentrations on liver function parameters. Future studies also should consider the temporal relationship between dietary intake and biospecimen collection in their design to improve the accuracy and reliability of the findings.

## 5 Conclusion

This population-based, cross-sectional study demonstrated that dietary selenium intake and blood selenium concentration impact liver function parameters in a population with high selenium intakes and blood concentrations and without major liver disturbances. Public health strategies should consider promoting moderate rather than excessive selenium intake as part of liver disease prevention efforts. Further research should also focus on the relationship between dietary selenium intake and blood selenium concentration with liver function in diverse populations to develop more precise prevention strategies.

## Data availability statement

The original contributions presented in the study are included in the article/supplementary material; further inquiries can be directed to the corresponding authors.

## Ethics statement

The studies involving humans were approved by the National Center for Health Statistics Research Ethics Review Board. The studies were conducted in accordance with the local legislation and institutional requirements. Written informed consent for participation was not required from the participants or the participants’ legal guardians/next of kin in accordance with the national legislation and institutional requirements.

## Author contributions

QL: Conceptualization, Writing – original draft. RH: Funding acquisition, Investigation, Writing – review & editing. ZP: Conceptualization, Supervision, Writing – review & editing. MZ: Conceptualization, Funding acquisition, Supervision, Writing – review & editing.
